# Supplementary material for: A pan-cancer analysis of homeobox family: expression characteristics and latent significance in prognosis and immune microenvironment
Source: Front Oncol. 2025 Feb 6;15:1521652. doi: 10.3389/fonc.2025.1521652 (PMC11840236; doi:10.3389/fonc.2025.1521652)
Supplement: Supplementary Figure 1 — Mutations of each HOX gene in 32 cancers (TCGA, PanCancer Atlas) by cBioportal. [file DataSheet1.zip › Suppl.files/raw code.zip!/code/4.survival/survivalplot.zip!/survivalplot/HOXA10_OS.PAAD.pdf]

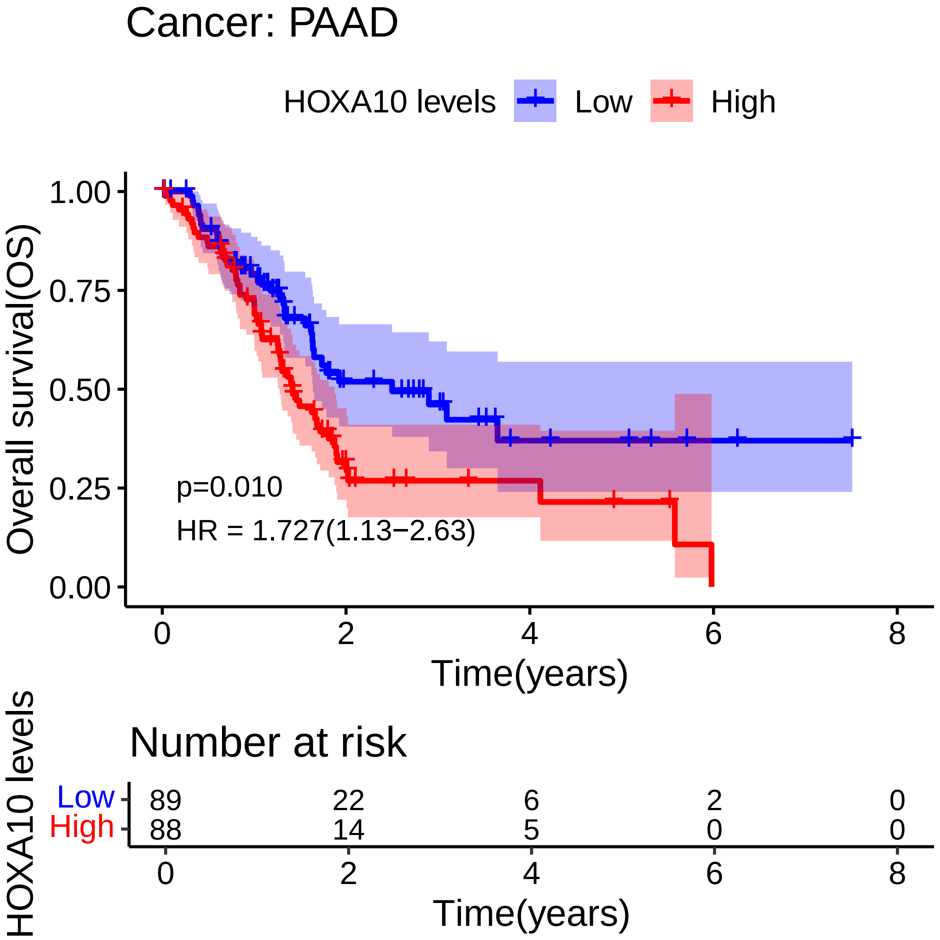
Cancer: PAAD
HOXA10 levels
Low
High
1.00
0.75
0.50
0.25
0.00
Overall survival(OS)
p=0.010
HR = 1.727(1.13−2.63)
0
2
4
6
8
Time(years)
Number at risk
HOXA10 levels
Low 89 22 6 2 0
High 88 14 5 0 0
0
2
4
6
8
Time(years)
